# Racial Disparities in Cataract Surgery Timeline and Intraocular Lens Selection: A Retrospective Study

**DOI:** 10.1167/tvst.12.11.20

**Published:** 2023-11-17

**Authors:** Seth E. Buscho, Ardalan Sharifi, Samir Cayenne, Yuanyi Zhang, Kevin H. Merkley, Praveena K. Gupta

**Affiliations:** 1 John Sealy School of Medicine, University of Texas Medical Branch, Galveston, Texas, USA; 2 Department of Ophthalmology & Visual Sciences, University of Texas Medical Branch, Galveston, Texas, USA; 3 Office of Biostatistics, University of Texas Medical Branch, Galveston, Texas, USA

**Keywords:** cataract, racial disparities, timeline, lens, premium, intraocular lens

## Abstract

**Purpose:**

There is a significant amount of literature focusing on racial inequities in utilization rates and intraoperative complications of cataract surgery. Unfortunately, little is known about racial disparities regarding the timeline of cataract surgery and intraocular lens (IOL) selection. This study investigated whether black patients have a different preoperative and postoperative cataract surgery timeline and IOL selection than white patients.

**Methods:**

A total of 10,235 patients (83.47% white) were retrospectively identified from a tertiary academic center who underwent cataract surgery between 2015 and 2022. Each patient's best corrected visual acuity (BCVA), slit lamp findings, and surgical timeline were recorded. IOL selection was categorized as standard or premium.

**Results:**

Black patients had significantly worse mean ± SD preoperative logMAR BCVA than white patients (0.47 ± 0.55 vs. 0.58 ± 0.70, respectively; *P* = 0.0117) and were significantly less likely to receive surgery within 120 days of referral (RR, 0.71 [95% confidence interval {CI}, 0.64-0.79]; *P* < 0.0001). White patients were 25%, 24%, and 29% less likely to follow-up than black patients at postoperative day 1, day 7, and day 30, respectively (*P* < 0.0001). White patients were 6.09 (95% CI, 3.49, 10.63) times more likely to receive a premium IOL compared to black patients (*P* < 0.0001).

**Conclusions:**

Black patients experienced more delays with receiving cataract surgery but are more adherent with postoperative follow-up. Black patients were far less likely to receive a premium IOL than white patients.

**Translational Relevance:**

Increased awareness of racial inequities in cataract surgery may improve the delivery of eye care to minority groups.

## Introduction

Despite efforts to improve eyecare delivery worldwide, there is growing concern surrounding healthcare inequities among minority groups in ophthalmology. With the onset of the COVID-19 pandemic, many pre-existing healthcare inequities in ophthalmology have only been exacerbated. Potential reasons for these inequities are multifactorial and likely reflect a combination of cultural barriers, economic factors (e.g., healthcare access, health insurance coverage, transportation), and systemic racial disparities in the delivery of eyecare.^1^ A significant amount of research regarding systemic inequities in eye care has shown that black patients are far less likely to have seen an eye care provider within the last year.^2^ Of the black patients who did utilize eyecare services, they less frequently attended outpatient encounters, had a greater than twofold higher rate of emergency department and inpatient encounters, and received less diagnostic testing during visits including optical coherence tomography or visual fields testing than white patients.^3^ These disparities in the delivery of eye care only compound with the fact that black patients carry a higher burden of many leading causes of blindness such as primary open-angle glaucoma, diabetic retinopathy, and cataract.^4^

Cataract is defined as an opacification of the lens secondary to aggregation of high molecular weight proteins or density variations in the lens secondary to lens fiber cell disarray and degeneration.^5^^,^^6^ Treatment of cataract involves extraction of the opacified lens and replacement with an artificial lens known as an intraocular lens (IOL). Recipients of this surgery are often very satisfied because it is a short outpatient procedure with a quick recovery that results in marked improvement in vision.^7^ Cataract extraction is one of the most frequently performed procedures because more than 20 million patients worldwide receive cataract surgery each year.^8^ Despite being a rapidly curable disease with surgical intervention, cataracts currently account for more than 65.2 million cases of visual impairment globally.^9^ One contributory factor to the high prevalence may be untimely or unequal delivery of surgical care to patients from minority groups. Indeed, it has been shown that unoperated senile cataracts accounted for 27% of cause-specific blindness among black patients, which was four times higher than the cause-specific prevalence of blinding cataracts in white patients.^10^ Most previous studies describing racial disparities amongst cataract surgery patients have focused on utilization rates of cataract surgery.^11^ The prior studies that did not focus on utilization rates of cataract surgery primarily explored intraoperative and postoperative complications of cataract surgery or readmission from cataract surgery among minority groups.^12^^,^^13^ Thus there is significant limitation in literature detailing racial disparities in cataract surgery beyond these factors.

In this study, we compared the preoperative and postoperative cataract surgery timeline, as well as the type of IOL implanted (e.g., standard vs. premium) for black versus white patients. Deviations from the recommended surgical timeline or hasty/ill-informed decisions made before surgery surrounding IOL selection may contribute to postoperative complications, visual disturbances, and residual refractive error, all leading to patient dissatisfaction. Previous studies have found that black patients experience significantly longer time-to-treatment for diabetic retinopathy^14^; however, the relationship regarding timeline of cataract surgery is not known. Additionally, many patients are presented with premium IOLs at their preoperative evaluation if they are deemed a suitable candidate, but oftentimes patients specifically request these IOLs. Most patients are satisfied with premium IOLs to achieve spectacle independence, despite some optical tradeoffs and the out-of-pocket costs.^15^ Based on our review of the English language ophthalmology literature, we believe there is no study that comprehensively evaluates the timeline of cataract surgery and assesses racial disparities regarding IOL selection among black and white patients.

## Materials and Methods

### Procedures

All protocol was approved by the University of Texas Medical Branch (UTMB Institution Review Board. Patient data were collected and maintained in accordance with Health Insurance Portability and Accountability Act guidelines. Because of the retrospective nature of the study, no patient consent was required.

### Patient Identification

This is a retrospective study that identified black and white patients who underwent cataract extraction with implantation of an IOL from January 1, 2015, to January 1, 2022, at the UTMB. A total of 11,984 were initially identified from the Epic electronic medical records database by CPT-10 code 66982 and 66984. Of these patients, four had unknown racial data, 10 were native Hawaiian/pacific islander, 50 were native American, 224 were Asian, 1832 were black, and 9864 were white. Because of the limited sample size of some racial groups, only data from black and white patients was used for statistical analysis. Both Hispanic and non-Hispanic patients were included in the black and white patient groups. Patients were excluded from this initial data pull if they did not receive a IOL after cataract extraction, the model or name of the IOL was not retrieved from the procedure note, or the patient underwent combined cataract surgery and surgery for another ophthalmic condition. After exclusion, our study consisted of 8543 white patients and 1692 black patients.

### Comorbidities and Confounding Factors

Each patient's cylinder that was used in our analysis was obtained at the preoperative appointment closest to the surgical date during IOL calculations. Because patients with regular astigmatism of >1.2 D, healthy corneas/retinas, and no chronic progressive ophthalmic diseases are considered candidates for premium IOLs, we defined patients with greater than 1.2 D of cylinder as “significant” astigmatism and patients with 1.2 D or less of astigmatism as “mild” or no astigmatism. The percent of patients with significant astigmatism, as well as basic demographic information including age, sex, ethnicity, and insurance status is presented in Table 1 and adjusted for in all subsequent analysis.

**Table 1.** Distribution of Patient Demographics, Insurance Status, and Cylinder < 1.2 D was Controlled for in Subsequent Analysis

Patient Demographic/Classification	White (n = 8543)	Black (n = 1692)	*P* Value
Age (yr), mean ± SD	70.36 ± 8.91	69.11 ± 9.80	<0.0001^a^
Sex			<0.0001^b^
Male	3738 (43.76%)	566 (33.45%)	
Female	4805 (56.24%)	1226 (66.55%)	
Ethnicity			<0.0001^b^
Hispanic	1816 (21.54%)	12 (0.72%)	
Not Hispanic	6616 (78.46%)	1645 (99.28%)	
Insurance			0.0309^b^
Commercial	1595 (18.67%)	292 (17.26%)	
County/Hospital District Authority	22 (0.26%)	6 (0.35%)	
Medicare/Medicaid	6754 (79.06%)	1375 (81.26%)	
Self-pay	172 (2.01%)	19 (1.12%)	
Cylinder			0.0015^b^
≤1.2 D	2162 (25.31%)	491 (29.02%)	
>1.2 D	6381 (74.69%)	1201 (70.98%)	

The *P* value was derived from comparing the means or distribution among the sample of black and white patients.

a The *t*-test.

b The χ^2^ test.

### Surgical Timeline Outcomes

Preoperative outcome measures for this study were the best-corrected visual acuity (BCVA) calculated as logarithm of the mean angle of resolution (logMAR), slit lamp findings (e.g., cataract type and grade) at the referral appointment, and the number of days between the referral appointment and surgical date (first of the two surgical dates in the case of patients with bilateral cataracts). The referral date was defined as the initial date at which the patient was recommended to receive cataract surgery by a UTMB provider and during which initial IOL calculations were made. The referral date was before standardized preoperative appointments for the cataract surgery. Patients were referred for surgery both by the UTMB University Eye Center (Galveston, TX, USA), as well as satellite UTMB ophthalmology clinics in the greater Galveston area. Postoperative outcome measures in this study were patient compliance with the recommended standardized follow-up appointments on postoperative day 1, day 7, and day 30, as well as postoperative BCVA obtained at the appointment closest to, but after, the standardized day 30 postoperative appointment.

### Intraocular Lens Selection Outcomes

The IOL each patient received was recorded and classified as either a “standard” or “premium” lens. In this study, a lens was considered “premium” if categorized as a toric IOL (to correct regular clinically significant corneal astigmatism (i.e., >1.2 D), an accommodating IOL (to enhance reading ability without glasses), a multifocal IOLs with either refractive or diffractive designs (to achieve additional focal points), or a multifocal toric IOL. Premium lens models were categorized based on the operative note written by the primary surgeon containing one of the models presented in Table 2 or any of the premium model names previously described.^16^

**Table 2.** Model Numbers for Categorization of Standard and Premium IOLs.

Classification (Standard/Premium)	IOL Model Numbers
Standard	SN60WF, SW60WF, SN60AT, ZCB00, MA60AC, MN60AC, MN60MA, MTA4U0, MTA5U0
Premium toric	SN6AT1, SN6AT2, SN6AT3, SN6AT4, SN6AT5, SN6AT6, SN6AT8, MX60ET
Premium multifocal	DFT015, SN6AD1, SV25T0, TFNT00
Premium multifocal toric	SND1T5, TFNT30, TFNT40, TFNT50, TFNT60

### Statistical Analysis

All statistical analysis was performed with SAS version 9.4. Numerical variables were compared using a Student's *t*-test when the distribution of data was normal or a Wilcoxon two-sample test when data normality was not present. The distribution of categorical variables was compared using a Chi square test and a Poisson regression was subsequently performed to identify the relative risk (RR) after adjusting for age, sex, ethnicity, insurance status, and significant astigmatism. The alpha value was set at 0.05 such that a *P* value <0.05 was considered statistically significant.

## Results

### Patient Presentation at Referral

To identify disparities in the timepoint at which white and black patients initially obtained vision care for their cataracts, we first obtained patient characteristics at the time they received their diagnosis and were referred for cataract surgery. Although white and black patients were diagnosed with cataracts of a similar grade, the visual acuity of white patients was significantly better than their black counterparts at the referral date. The mean logMAR (Snellen fraction [SD]) visual acuity for white patients was 0.47 (20/59 [0.55]) compared to 0.58 (20/76 [0.70]) for black patients (*P* = 0.0117) (Table 3). White and black patients were also found to have significantly different types of cataracts. White patients were more likely to have a nuclear sclerosis cataract while black patients were more likely to have cortical, posterior subcapsular cataracts, and mixed cataracts (*P* < 0.0001).

**Table 3.** Patient Characteristics at the Time of Diagnosis

Clinical Characteristic	White	Black	*P* Value
BCVA (LogMAR), mean ± SD	0.47 ± 0.55	0.58 ± 0.70	0.0117^a^
Cataract grade, mean ± SD	2.40 ± 0.83	2.38 ± 0.83	0.5258^b^
Cataract Type			<0.0001^c^
NSC	5188 (89.13%)	1220 (82.49%)	
CC	185 (3.18%)	137 (9.26%)	
PSC	287 (4.93%)	72 (4.87%)	
Mixed	140 (2.41%)	45 (3.04%)	
Other	21 (0.36%)	5 (0.34%)	

Best corrected visual acuity and cataract grade were recorded for the worst eye in white and black patients. Patient's cataracts were classified as nuclear sclerosis (NSC), cortical (CC), posterior subcapsular (PSC), mixed, or other.

a Wilcoxon two-sample test, N = 1506-5880.

b The *t*-test, N = 1361-5369.

c The χ^2^ test, N = 1479-5821.

### Postoperative Timeline and Outcomes

**Figure 1.**
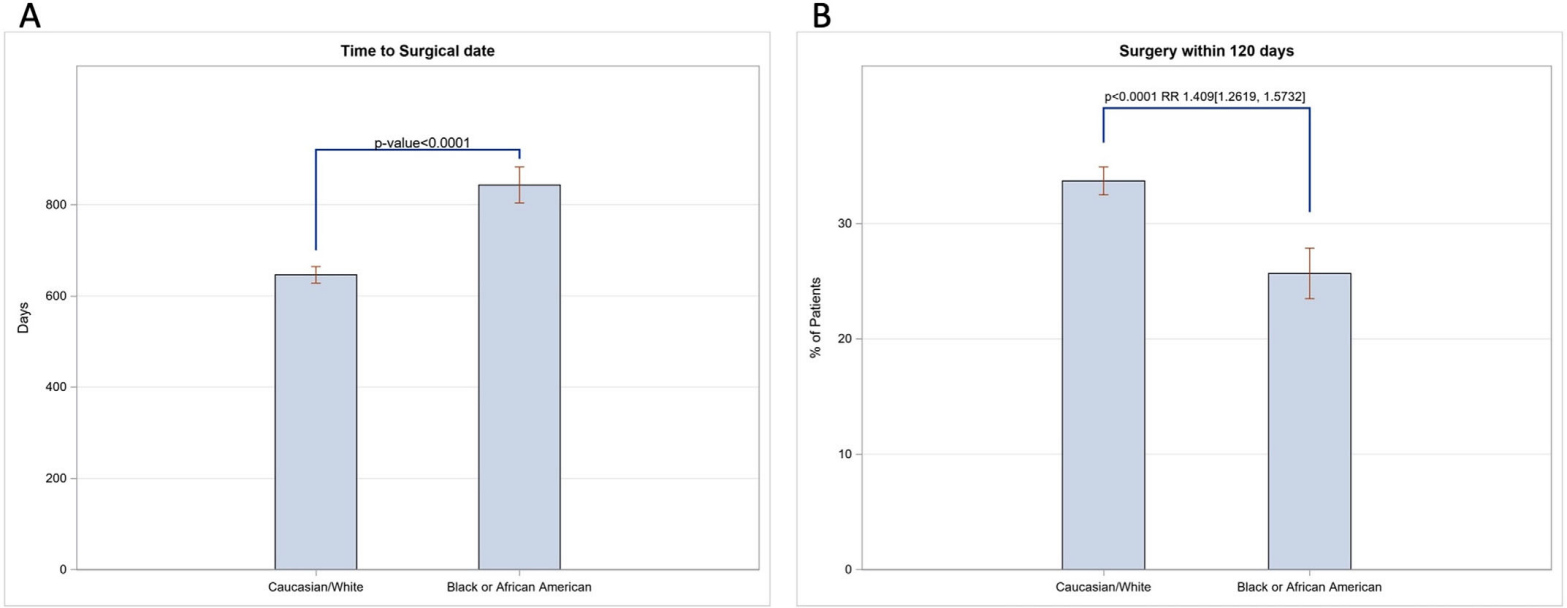
(**A**) Recorded number of days between the referral date for cataract surgery and the surgical date for white versus black patients. Wilcoxon two-sample test, N = 1519–5887. (**B**) Percent of white and black patients who received surgery within 120 days of the referral date. The χ^2^ test, N = 1692–8543; RR from poison regression after adjustment for age, sex, ethnicity, insurance status, and astigmatism.

White patients had a surgical date a mean of 646.08 (95% confidence interval [CI], 628.07-664.08) days after the referral date and black patients had a surgical date 843.20 (95% CI, 803.81-882.59) days after the referral date (Fig. 1A). Similarly, 1984 (33.70%) white patients received surgery within four months of their referral date, but 390 (25.67%) black patients received surgery within this same timeframe. Overall, white patients were found to be 1.292 (95% CI, 1.1612-1.4375) times more likely to receive surgery within 120 days as compared to black patients without adjusting for confounders, or 1.41 (95% CI, 1.26-1.57) times more likely to receive surgery within 120 days after adjusting for age, sex, ethnicity, insurance status, and astigmatism (*P* < 0.0001) (Fig. 1B).

**Figure 2.**
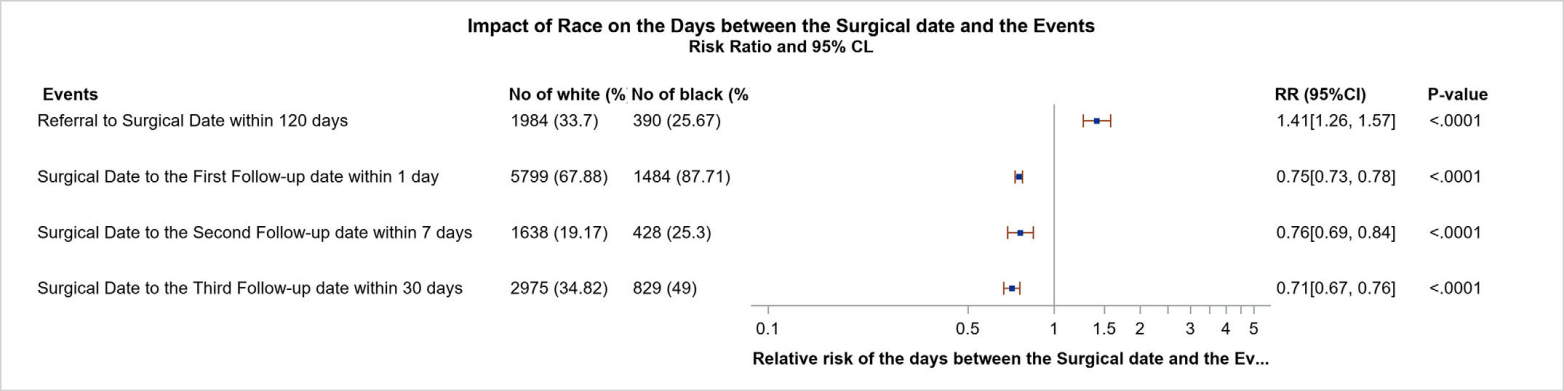
Forest plot depicting the RR that white versus black patients received surgery within 120 days and attended their postoperative day 1, day 7, and day 30 follow-up appointments. N = 1692–8543.

The postoperative timeline is scheduled to be consistent for all patients such that follow-up occurs at one day, one week, and one month after the surgical date. Of the 8,543 white patients who received cataract surgery, 5799 (67.88%) were adherent with their postoperative day 1, 1638 (19.17%) were adherent with their postoperative day 7, and 2975 (34.82%) were adherent with their postoperative day 30 appointments. In contrast, 1484 (87.71%), 428 (25.30%), and 829 (49.00%) of the 1692 black patients who underwent cataract surgery attended their postoperative day 1, day 7, and day 30 appointments, respectively (*P* < 0.0001). Overall, we found that white patients were 25% less likely to follow-up at one day, 24% less likely to attend their postoperative day 7 appointment, and 29% less likely to attend their third follow-up appointment on day 30 (Fig. 2).

After cataract surgery, white patients experienced better BCVA compared to black patients. White patients had a mean logMAR (Snellen fraction [SD]) visual acuity of 0.16 (20/28 [0.24]) as compared to 0.19 (20/30 [0.29]) for black patients (*P* < 0.0001).

### IOL Selection

**Figure 3.**
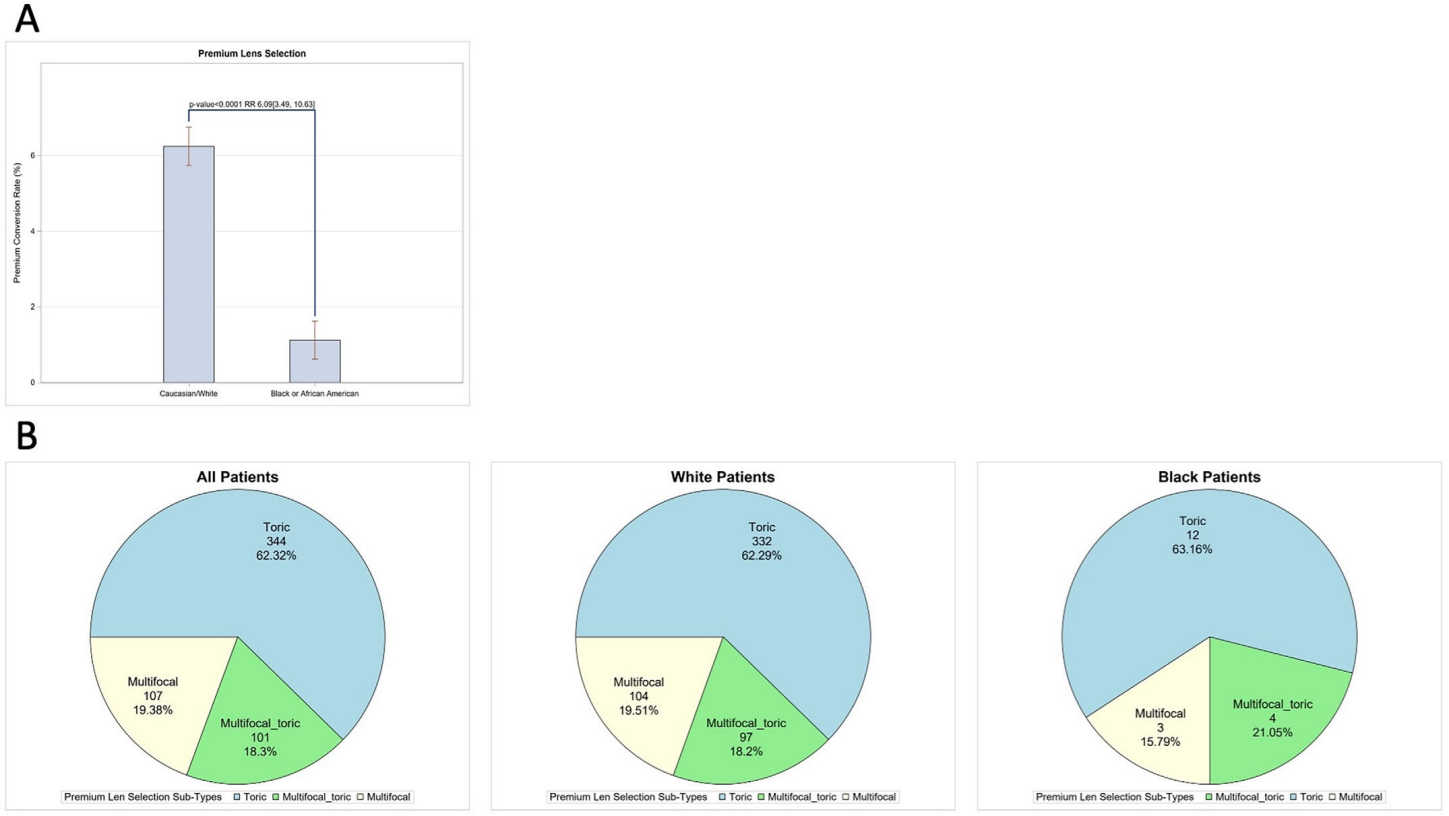
(**A**) Percent of white versus black patients who received a premium IOL during cataract surgery. The χ^2^ test, N = 1692–8543; RR from Poisson regression adjusted for age, sex, ethnicity, insurance status, and astigmatism. (**B**) Distribution of premium IOLs that all patients, only white patients, and only black patients received.

Selecting an IOL is one of the most important decisions a patient can make before receiving cataract surgery. Premium IOLs allow some patients to achieve spectacle independence and high visual acuity at many focal points, which can be very gratifying. Most patients in this study received a standard IOL; however, 552 (5.39%) patients in this study elected to have a premium IOL implanted. When assessing racial differences in premium IOLs, we found that the premium conversion rate for white patients was 6.24% compared to 1.12% for black patients, indicating that white patients were 5.11 (95% CI, 2.98-8.76) times more likely to receive a premium IOL (*P* < 0.0001). After adjusting for age, sex, ethnicity, cylinder, and insurance status, white patients were 6.09 (95% CI, 3.49-10.63) times more likely to receive a premium IOL (Fig. 3b).

Of the 552 patients who received premium IOLs, 344 received toric IOLs, 107 received multifocal IOLs, and 101 received multifocal toric IOLs. No statistically significant differences were found when comparing the distribution of premium IOL subtypes among racial groups (*P* = 0.8928) (Fig. 3b).

## Discussion

The results of this study demonstrate that black patients are significantly more likely to be referred for cataract surgery at a lower visual acuity and receive surgery at later timepoint after referral for cataract surgery compared to white patients. Contrary to our hypothesis, black patients were more likely to attend their postoperative follow-up appointments on postoperative day 1, day 7, and day 30. Black patients were far less likely to receive a premium IOL than their white counterparts, although the subtypes of premium IOLs that they received were not significantly different than white patients. Overall, our study is novel in that it shows black patients currently face significant hurdles in both receiving timely cataract surgery and achieving spectacle independence from premium IOLs.

Delayed cataract surgery and prolonged wait times are associated with progressive loss of vision, reduced quality of life, and increase risk of falls.^17^ Indeed, it has been demonstrated that expedited first-eye cataract surgery improves levels of physical activity, confidence, and reduces levels of anxiety and depression.^18^ In this study we found that white patients were 1.30 (95% CI, 1.13-1.45) times more likely to receive surgery within 120 days of referral. In line with these data, one study by Wu et al.^19^ found that black race versus white race was associated with significantly lower odds (adjusted odds ratio [OR] = 0.79 [95% CI, 0.77-0.81]) of receiving cataract surgery within one year for their Veterans Health Administration population^19^; however, black Medicare patients had a similar likelihood of receiving cataract surgery within one year compared to white Medicare patients (unadjusted OR = 1.01 [95% CI, 0.99-1.03] vs. adjusted OR = 1.03 [95% CI, 1.01-1.05]). Another study by Broman et al.^20^ found that U.S. Hispanic individuals were 3.87 times more likely to have visually significant cataracts (BCVA worse than 20/40) compared with white individuals.^20^ Collectively, our results, together with the studies by Wu et al.^19^ and Broman et al.,^20^ suggests that racial minorities face undue burdens in both being referred for cataract surgery and having their cataract extracted. Because the patients in this study were evaluated by time since referral rather than time since diagnosis, it is likely that many patients (especially black patients) were tolerant of cataract-related vision impairment for a long period of time.

Although black patients experience undue delays in being diagnosed with cataracts and receiving cataract surgery, they had better follow-up compared to white patients after receiving surgery. To the best of our knowledge, there are currently no studies that have evaluated postoperative compliance after cataract surgery among black and white patients. A previous study published by Parvus and colleagues,^21^ however, found that black patients had significantly greater post-operative follow-up after surgery for macular hole than white patients. A total of 77.2% of black patients in their study followed up six months after surgery compared to 73.2% of white patients, and this relationship was consistent at 12 months after the surgical date. Although the causative factors for this relationship have yet to be elucidated, it is possible that white patients were less inclined to attend postoperative appointments than black patients because they had a superior mean logMAR postoperative visual acuity. Postoperative evaluation multiple times within one month of cataract surgery is critical to patient compliance with recovery instructions and medications, identify complications, and ensure proper healing, and inadequate follow-up has been associated with higher rates of postoperative sequelae and uncorrected residual refractive error.^22^^,^^23^

Current intraocular lens offerings include an expanding array of options including accommodating, multifocal, toric, and multifocal toric IOLs. These innovative lens designs allow patients to have multiple functional focal points and correct astigmatism with increasing accuracy while minimizing unwanted aberrations or dependency on prescriptive lenses. In this study, we observed that white patients were 5.11 (95% CI, 2.98-8.76) times more likely to receive an advanced optics IOL than black patients, but the distribution of advanced IOL subtypes was not different among individual racial groups. The IOL that the patient ultimately selects is a complex decision between the operating ophthalmologist and the patient themselves. First, the ophthalmologist must determine the patient's motivation to tolerate corrective glasses/contacts and their desire to be spectacle-free. The patient must also demonstrate healthy corneas and retinas to be good candidates for advanced IOL optics. Toric IOLs are generally presented as an option for patients who have regular corneal astigmatism of greater than 1.2 D and whose astigmatism causes subjective visual impairment. Finally, it is the responsibility of the ophthalmologist to make an IOL recommendation that aligns with the patient's goals, career, hobbies, daily activities, and financial capabilities.

It is possible that any differences in premium IOL conversion rates could arise from a multitude of factors related primarily and secondarily to racial group, so assigning causation is complex. One of the many potential causes is implicit bias, in which a provider uses time-saving heuristics that are detrimental to the medical care of minorities.^24^ It is also possible that the discrepancies could be due to communication barriers from minority communities having a degree of mistrust regarding newly developed medical operations.^25^^,^^26^ We suspect, though, that the primary factor in premium IOL selection is financial limitation from longstanding socioeconomic disparities, which we did not control for in this study. Socioeconomic limitations impact access to care, for instance with transportation and educational opportunities, which creates barriers for patients to have equal access to these technologies. Further studies should look closer at disparities among patients who have adopted premium IOLs at the time of cataract surgery to determine causation.

The findings from this study have important and immediate applicability to the clinical setting. Ophthalmologists and optometrists should be mindful of these disparities when providing future eye care to patients from minority groups and maintain awareness of any implicit bias they may have during preoperative assessments. Increased emphasis should be placed on educating black patients about seeking medical care quickly as visual symptoms arise. Moreover, some patients may be unaware of the common symptoms of cataract which could lead to delays in seeking eye care or recognition by the patient's provider. Finally, premium IOLs offer a unique opportunity for many patients to regain spectacle independence and achieve increased visual acuity at more than one focal point. Ophthalmologists should be mindful of these racial disparities in IOLs, make an increased effort to provide education to patients unaware of premium IOLs, and encourage black patients who are strongly considering premium IOLs if they are a good candidate.

### Strengths and Weaknesses

Strengths of this study include its large sample size with a sample population representative of the general population. Patients identified in this study came from urban, suburban, and rural backgrounds and had numerous forms of payment methods including self-pay, private commercial insurance, hospital district authority, and government insurance (e.g., Medicare/Medicaid). An additional strength of the study is that we controlled for pre-existing astigmatism when calculating the RR that white patients would receive a premium IOL compared to black patients. The primary weaknesses of this study are inherent to its retrospective nature. First, we were unable to control for the income status of patients in this study because of limited financial information available in medical records. A second weakness of the study is that we did not determine reasons that patients failed to attend their postoperative visits.

## Conclusions

This study's findings have important implications regarding racial disparities in the timeline of cataract surgery and intraocular lens (IOL) selection. Black patients have longer wait times for cataract surgery compared to their white patient counterparts, but they also adhere to post-operative follow-up instructions more closely. Despite their close adherence, black patients suffered from worse postoperative BCVA. The study also shows a considerable disparity in the choice of premium IOLs, with white patients being substantially more likely than black patients to receive premium IOLs. These findings highlight the need for addressing racial disparities in eye care access and decision-making, with focus on equitable eye care for all patients.
